# Impact of Glyme Ether Chain Length on the Interphasial Stability of Lithium‐Electrode in High‐Capacity Lithium‐Metal Battery

**DOI:** 10.1002/advs.202404245

**Published:** 2024-06-21

**Authors:** Arghya Dutta, Kyosuke Matsushita, Yoshimi Kubo

**Affiliations:** ^1^ Center for Green Research on Energy and Environmental Materials National Institute for Materials Science 1‐1 Namiki Tsukuba 305‐0044 Japan; ^2^ Battery Research Platform Center for Green Research on Energy and Environmental Materials National Institute for Materials Science 1‐1 Namiki Tsukuba 305‐0044 Japan; ^3^ NIMS‐SoftBank Advanced Technologies Development Center National Institute for Materials Science 1‐1 Namiki Tsukuba 305‐0044 Japan

**Keywords:** glyme‐ether‐electrolyte, Li deposition, Li metal battery, morphological analysis, solid‐electrolyte‐interphase

## Abstract

The realization of lithium‐metal (Li) batteries faces challenges due to dendritic Li deposition causing internal short‐circuit and low Coulombic efficiency. In this regard, the Li‐deposition stability largely depends on the electrolyte, which reacts with Li to form a solid electrolyte interphase (SEI) with diverse physico‐chemical properties, and dictates the interphasial kinetics. Therefore, optimizing the electrolyte for stability and performance remains pivotal. Hereof, glyme ethers are an emerging class of electrolytes, showing improved compatibility with metallic Li and enhanced stability in Li─Air and Li─Sulfur batteries. Yet, the criteria for selecting glyme solvents, particularly concerning Li deposition and dissolution processes, remain unclear. The SEI characteristics and Li deposition/dissolution processes are investigated in glyme‐ether‐based electrolytes with varying chain lengths, using lithium bis(trifluoromethanesulfonyl)imide (LiTFSI) and lithium nitrate (LiNO₃) salts under high capacity and limited electrolyte conditions. Longer glymes led to more homogeneous SEI, particularly pronounced with LiNO₃, minimizing surface roughness during stripping, and promoting compact Li deposits. Higher reductive stability, resulting in homogeneous interphasial properties, and slower kinetics due to high desolvation barrier and viscosity, underline stable Li growth in longer glymes. This study clarifies factors guiding the selection of glyme ether‐based electrolytes in Li metal batteries, offering insights for next‐generation energy storage systems.

## Graphical abstract

Although glyme ethers show enhanced compatibility with lithium (Li) electrode and better stability in Li─Air and Li─Sulfur batteries, the selection criteria for glyme solvents remain unclear. Investigation of Li deposition/dissolution in varying glyme ethers reveals that longer chain glymes lead to compact Li growth and homogeneous interphase, impacting battery performance. This study aids in glyme ether selection for advanced energy storage.

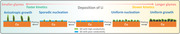

## 1 Introduction

Lithium (Li) metal is considered a highly promising negative electrode material for next‐generation high‐energy rechargeable batteries due to its exceptional specific capacity (3860 mAh g^−1^), low electrochemical potential (−3.04 V versus standard hydrogen electrode (SHE)), and low density (0.534 g cm⁻^3^).^[^ ^1^, ^2^, ^3^ ^]^ Combining Li metal negative electrodes with high‐capacity positive electrode materials like air or sulfur (S) is promising for advancing high‐energy‐density energy storage systems.^[^ ^4^, ^5^, ^6^, ^7^ ^]^ However, two major challenges in charging lithium‐metal batteries (LMBs) are the dendritic growth of deposited Li, which can lead to internal short‐circuits, and low Coulombic efficiency (CE). The low CE is caused by non‐compact/porous Li becoming electrically disconnected during stripping/plating cycles, as well as the depletion of active Li due to its high reactivity with nonaqueous electrolytes.^[^ ^8^, ^9^, ^10^ ^]^ As a result, Li‐deposition stability largely depends on the electrolyte, which reacts with Li to form a solid electrolyte interphase (SEI) with diverse physical and chemical properties, and dictates the interphasial kinetics.^[^ ^11^, ^12^, ^13^, ^14^, ^15^, ^16^, ^17^, ^18^, ^19^ ^]^ Therefore, optimizing the electrolyte for the stability and performance of LMBs remains pivotal.^[^ ^20^ ^]^

While traditional carbonate electrolytes, commonly used in Li‐ion batteries, exhibit poor stability when in contact with Li metal and positive electrodes like air, recent attention has shifted towards glyme‐ether‐based electrolytes due to their enhanced compatibility with metallic Li and improved stability, particularly in Li─Air and Li─S batteries.^[^ ^21^, ^22^, ^23^, ^24^, ^25^, ^26^, ^27^, ^28^ ^]^ Although considerable research has been devoted to understanding the physical properties of glyme‐based electrolytes, developing new ether solvents for improved Li‐metal compatibility, and elucidating the failure mechanism of Li‐metal electrodes, a comprehensive understanding of the criteria for selecting simple commercial glyme solvents and establishing a systematic correlation between glyme chain length and Li deposition and dissolution processes, SEI formation, and overall cell performance remains elusive.^[^ ^29^, ^30^, ^31^, ^32^, ^33^, ^34^, ^35^, ^36^, ^37^ ^]^ The physicochemical properties of the glymes, including viscosity, Li⁺‐ion solvation, ionic conductivity, and electrochemical stability, vary significantly based on the chain length of glyme ethers.^[^ ^38^, ^39^ ^]^ Additionally, the choice of Li salts employed plays a crucial role in modulating these electrolyte characteristics.^[^ ^40^ ^]^ The complex interplay among all these electrolyte properties affects the electrode kinetics as well as the interphasial properties, which influence the Li deposition/dissolution processes.^[^ ^41^ ^]^ Therefore, despite the growing interest in glyme‐ether‐based electrolytes in LMBs, the large variations in the physical properties and (electro)chemical stabilities of the electrolytes collectively make it challenging to establish definitive selection criteria for glyme ethers. Hence, there exists a critical importance to address these knowledge gaps to drive forward the development of high‐performance energy storage systems.

In this study, we undertook a comprehensive investigation to systematically explore the characteristics of the SEI, interphasial kinetics, and the morphologies of Li deposition/dissolution in glyme‐ether‐based electrolytes with varying chain lengths, and their impact on the cycle‐life of the cell. We specifically investigated electrolytes containing a Li⁺ concentration of 1 m with two widely utilized Li salts in Li─Air and Li─S batteries: either lithium bis(trifluoromethanesulfonyl)imide (LiTFSI) or lithium nitrate (LiNO₃). To meet the practical requirements of high energy density cells, our experimental design involved cell cycling under conditions of high areal‐capacity of 4 mAh cm⁻^2^, limited electrolyte content of 5–6 g Ah^−1^, and a current density of 0.2 mA cm⁻^2^, unless otherwise mentioned. Our research outcomes unveiled noteworthy trends: longer chain‐length glymes demonstrated superior cycle life, regardless of the Li salt used. However, our comparison of Li salts indicated that LiTFSI exhibited inferior performance compared to LiNO₃ with any glyme used. These observed outcomes are attributed to the differences in the solvation structure depending on the glyme chain length and the electrolyte salt, which influence the deposition/dissolution kinetics, and the propensity for electrolyte decomposition, resulting in varied SEI properties. Overall, this research elucidates critical parameters influencing glyme ether‐based electrolytes in LMBs, providing valuable insights for advancing the next‐generation energy storage systems.

## 2 Results

### 2.1 Galvanostatic Li Stripping/Plating Cycles

**Figure 1:**
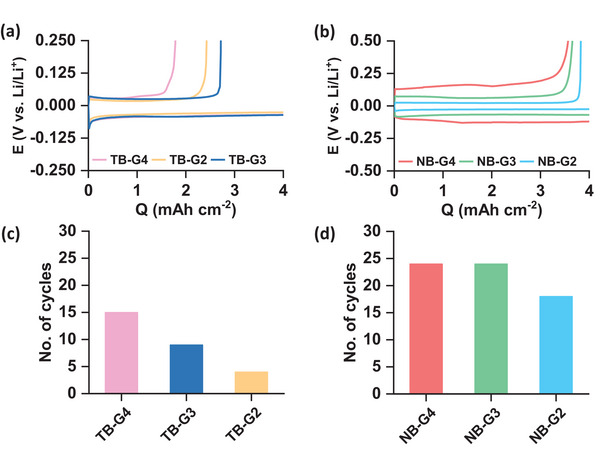
Galvanostatic voltage profiles for Li stripping/plating in Li|Cu cells at 0.2 mA cm⁻^2^ current density using a) LiTFSI and b) LiNO₃‐based electrolytes. c,d) Cycling stability of the same cells.

Li|Cu cells (schematically shown in Figure S1, Supporting Information) were subjected to cycling experiments using various electrolytes containing either 1.0 m LiTFSI or 1.0 m LiNO₃, both supplemented with 0.05 m lithium bromide (LiBr) as an additive in diethylene glycol dimethyl ether (diglyme or G2), triethylene glycol dimethyl ether (triglyme or G3), and tetraethylene glycol dimethyl ether (tetraglyme or G4) solvents. These are the widely used electrolytes in Li─Air and Li─S batteries, where the Li metal negative electrode is required to endure cycling at high capacity.^[^ ^42^, ^43^, ^44^, ^45^, ^46^ ^]^ The incorporation of LiBr in the electrolytes is a well‐established strategy aimed at reducing the high charge overpotential in Li─Air batteries and facilitating the stable deposition and dissolution of Li metal.^[^ ^11^, ^12^, ^40^, ^45^ ^]^ All the cells underwent cycling, operating at 0.2 mA cm⁻^2^ current density and an areal capacity of 4 mAh cm⁻^2^, while employing a limited quantity of electrolyte measuring ≈ 5–6 g Ah^−1^. **Figure** **1**a illustrates the voltage profiles corresponding to the first‐cycle Li stripping and plating processes of the Li|Cu cells, employing 1.0 m LiTFSI + 0.05 m LiBr electrolytes in G2, G3, and G4 solvents (referred to as TB‐G2, TB‐G3, and TB‐G4, respectively). Notably, all cells exhibited a relatively low first‐cycle CE within the range of ≈25% to 70%. Figure 1b presents similar voltage profiles for 1.0 m LiNO₃ + 0.05 m LiBr electrolytes with G2, G3, and G4 solvents (denoted as NB‐G2, NB‐G3, and NB‐G4, respectively). Interestingly, within these LiNO₃‐based electrolytes, the first‐cycle CE remained within the range of ≈75% to 95%. A comparative analysis of the voltage profiles revealed two notable distinctions when employing different glymes with LiTFSI and LiNO₃ salts. First, LiTFSI and shorter glyme electrolytes exhibited reduced voltage polarization attributed to their higher ionic conductivities, as shown in Figure S2 (Supporting Information). Second, LiTFSI‐based electrolytes exhibited lower CE. However, it is important to highlight that there was no consistent and clear trend in the first‐cycle CE based on the length of the glyme chain. In fact, repeated experiments revealed significant variability in the first‐cycle CE for each glyme, especially in LiTFSI electrolytes.

Figure 1c,d present the number of cycles for the LiTFSI and LiNO₃‐based electrolytes, respectively. Notably, as depicted in the voltage versus time curves in Figures S3 and S4 (Supporting Information), all cells experienced failures due to short‐circuits, with more pronounced occurrences observed in LiTFSI‐based electrolytes. As observed in Figure 1c,d, the cycling stability trends for both LiTFSI and LiNO₃‐based electrolytes follow the same order: G2 < G3 < G4. Besides, these findings unequivocally establish that cycling stability is superior in LiNO₃‐based electrolytes compared to LiTFSI‐based electrolytes, irrespective of the glyme solvent employed. Based on these results, we then compared the cycling stabilities of Li‐O₂ cells using NB‐G2, NB‐G3, and NB‐G4 electrolytes. The galvanostatic voltage profiles are shown in Figures S5–S8 (Supporting Information) display the capacity versus cycle number plots. Similar to the Li|Cu cells, the cycling stability of the Li‐O₂ cells also followed the order NB‐G2 < NB‐G3 < NB‐G4. More importantly, the cycling of the cells with NB‐G2 and NB‐G3 ended due to short‐circuit during prolonged cycling. While the cycle life of Li‐O₂ cells largely depends on the stability of the positive electrode and needs further investigation on the effects of glyme chain length, the advantage of using longer glymes concerning the negative electrode is evident.

### 2.2 Morphologies of the Electrodes during Cycling

**Figure 2:**
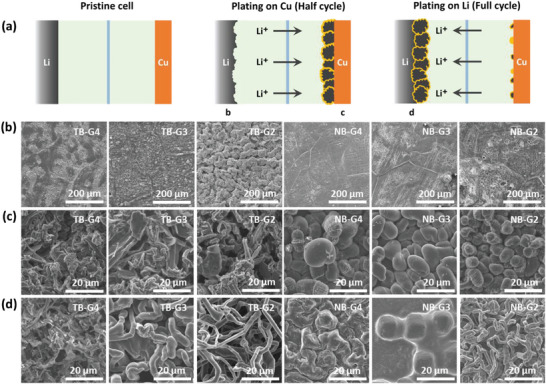
a) Schematic representation of the different stages of Li stripping/plating in a Li|Cu cell. b) Morphologies of Li metal surface after stripping of 4 mAh cm⁻^2^ Li at a current density of 0.2 mA cm⁻^2^. Morphologies of plated Li on (c) Cu surface and (d) Li surface that was stripped in the previous half cycle.

Since all the cells experienced short‐circuit during cycling, we examined the morphologies of both the Li and Cu electrodes using a scanning electron microscope (SEM) during stripping and plating processes. **Figure** **2**a schematically illustrates the various stages of the very first stripping/plating process. This process starts with the stripping of the Li electrode and the plating on the Cu electrode during the first half cycle, followed by the stripping from the Cu electrode and the plating back on the Li electrode during the complete cycle. Figure 2b shows the morphologies of the Li electrodes after 4 mAh cm⁻^2^ stripping in various electrolytes. Stripping in LiTFSI‐based electrolytes resulted in notable surface inhomogeneity spanning several micrometers to tens of micrometers on the Li metal surface. Conversely, the extent of physical damage to the electrode surface was considerably lower in LiNO₃‐based electrolytes. Nonetheless, the surface roughness increased in both cases as the glyme chain length decreased.

The morphologies of the Li deposits on the Cu electrodes are illustrated in Figure 2c. The observed morphologies of Li are notably influenced by the choice of Li salt and can be categorized into two distinct types. LiTFSI promotes a mixed morphology of moss and whisker‐like deposits, whereas spherical morphologies are formed in LiNO₃‐based electrolytes. In the case of TB‐G4, a relatively smaller number of long whisker‐like Li deposits are embedded within the moss‐like Li deposits throughout the electrode. Conversely, in TB‐G2 and TB‐G3, besides the coexistence of two morphologies at the same location (Figure 2c), whisker and moss‐like morphologies grow separately in different locations (Figures S9–S12, Supporting Information). From these observations, it is inferred that these smaller glymes, G2 and G3, exhibit a higher tendency to form whisker‐like Li compared to G4. In LiNO₃‐based electrolytes, glyme‐dependent morphological differences are also observed in the deposited Li. Upon closer examination of Figure 2c, variations in the dimensions of the deposited Li are evident, depending on the glyme solvent used with LiNO₃. Specifically, as the length of the glyme decreases, the spherical deposits become smaller.

Figure 2d illustrates the morphologies of Li deposited on the previously stripped Li electrodes. Interestingly, a considerable variation in Li morphologies is observed in this scenario too, which cannot be readily attributed solely to a specific type of Li salt. Particularly, in the case of TB‐G4, deposits with mixed morphology, similar to the case of Cu electrode, are observed. In contrast, both TB‐G3 and TB‐G2 exhibit distinctive whisker‐like deposits, with the whiskers appearing thinner in the case of TB‐G2. For NB‐G4 and NB‐G3, compact spherical deposits are observed, consistent with the observation on the Cu electrode. However, the morphology of deposited Li on the stripped Li electrode in NB‐G2 differs notably from that on the Cu electrode and shows both whisker‐like and spherical (Figure S13, Supporting Information) morphologies in different locations.

These morphological investigations of the Li and Cu electrodes reveal several important observations: as the glyme chain length decreases, the roughness of the Li electrode increases during stripping, and morphologies of the deposited Li on the flat Cu surface show little variation with increasing tendency towards forming whiskers in LiTFSI, but plating on the previously stripped Li shows a clear correlation with glyme dependent prior roughness. The propensity towards forming whisker‐like/dendritic Li on previously stripped Li surface increases with decreasing glyme chain length. These differences in morphological trends of deposited Li on Cu and previously stripped Li surfaces underscore the significant influence of not only the physical properties of electrolytes but also the interphasial properties of the electrode on deposition morphologies. Nevertheless, it is essential to recognize that the properties and composition of the electrolyte are crucial in determining the interphasial properties of the electrode. Therefore, it is imperative to achieve a comprehensive understanding of how electrolyte composition influences interphasial kinetics, SEI formation, and the deposition process to establish correlations between electrolyte type, Li deposition morphology, and cell performance.

### 2.3 Interpretations of the Electrolyte‐Dependent Morphologies

#### 2.3.1 Stripping from Li

**Figure 3:**
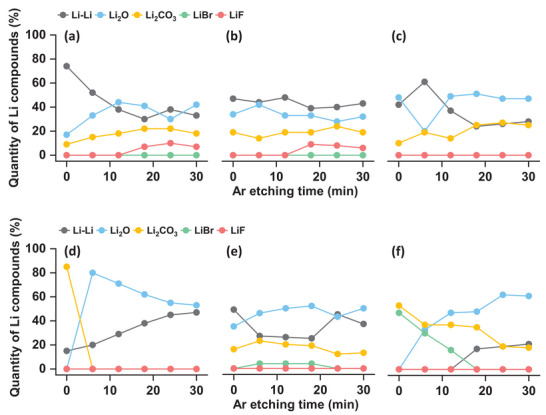
Quantification of Li compounds by X‐ray photoelectron spectroscopy (XPS) at different depths of the Li electrode surface after stripping (4 mAh cm⁻^2^ at a current density of 0.2 mA cm⁻^2^) in (a) TB‐G4, (b) TB‐G3, (c) TB‐G2, (d) NB‐G4, (e) NB‐G3, and (f) NB‐G2.

Our investigation initially focuses on exploring the variations in Li electrode morphologies during stripping, which are influenced by the electrolyte composition. When a current is applied, Li⁺ cations migrate through the SEI layer and dissolve in the electrolyte. The pristine Li surfaces consist of a native layer, formed during manufacturing and storage, along with an SEI layer developed upon exposure to the electrolyte.^[^ ^47^, ^48^, ^49^ ^]^ Ideally, this surface layer should exhibit homogeneity, good Li⁺ ion conductivity, and low electronic conductivity.^[^ ^50^ ^]^ However, in reality, both physical and chemical irregularities contribute to spatially varying conducting properties and uneven current distribution within the surface layer.^[^ ^47^, ^48^, ^50^ ^]^ These irregularities result in the formation of “hot spots”, which are areas with enhanced Li⁺ conductivity, facilitating the rapid dissolution of Li from those regions. Once sporadic dissolution starts, further removal of Li from adjacent sites occurs for two primary reasons: first, removing Li away from the initially formed Li vacancy is energetically less favorable, and second, Li⁺ transport through the SEI is more efficient in those regions.^[^ ^51^ ^]^ Consequently, continuous stripping from the same site leads to void formation beneath the SEI, causing its collapse and exposing fresh Li surface, thereby triggering further dissolution.^[^ ^49^, ^52^ ^]^ This self‐amplifying process eventually leads to the formation of pits and large surface‐inhomogeneity. Moreover, if the rate of Li dissolution is high, the preferential Li⁺ transport through “hot spots”, and subsequent pit formation are expected to be exacerbated. **Figure** **3**a–f depicts the quantitative analysis of Li compounds within the surface layer after the stripping of Li electrodes under varying electrolyte conditions. These compounds were estimated by analyzing different components of the Li1s X‐ray photoelectron spectra (XPS), acquired at various depths through argon (Ar) etching for 0, 6, 12, 18, 24, and 30 min. The XPS spectra for the relevant elements obtained from different electrodes are shown in Figures S14–S19 (Supporting Information). Results in Figure 3a–c reveal that after stripping in LiTFSI‐based electrolytes with any glyme, the surface layer exhibits significant chemical heterogeneity, with no clear trend dependent on the glyme type. Across all cases, the layer primarily comprises lithium oxide (Li₂O) and lithium carbonate (Li₂CO₃), in addition to metallic Li. Conversely, while NB‐G2 and NB‐G3 exhibit similar inhomogeneity within the surface layer, NB‐G4 displays a notably more uniform layer predominantly consisting of Li₂O and Li, as illustrated in Figure 3d–f. Although these data do not establish a clear trend, it is apparent that the use of the longest glyme, G4, yields enhanced chemical homogeneity when paired with LiNO₃ as the electrolyte salt and the ratio of Li₂O to Li₂CO₃ decreases with decreasing glyme chain length in the order, NB‐G4 > NB‐G3 > NB‐G2. To directly observe the compositional inhomogeneities on the Li electrode surface, we performed energy dispersive X‐ray (EDX) mapping after stripping in NB‐G2, NB‐G3, and NB‐G4 electrolytes and the results are shown in Figures S20–S22 (Supporting Information). The uneven distribution of oxygen and carbon elements on the Li surface provides clear evidence of the nonuniform distribution of SEI products. The EDX results in Table S1 also indicate that the oxygen and carbon contents increased as the glyme length decreased. These observed inhomogeneities suggest that sporadic stripping from preferred locations is feasible.

**Figure 4:**
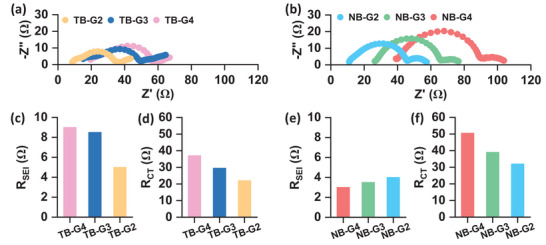
Nyquist plots of Li|Li symmetric cells using a) LiTFSI‐based and b) LiNO₃‐based electrolytes. Interphasial resistance (R_SEI_) for c) LiTFSI and e) LiNO₃‐based electrolytes. Charge‐transfer resistance (R_CT_) for d) LiTFSI and f) LiNO₃‐based electrolytes.

**Figure 5:**
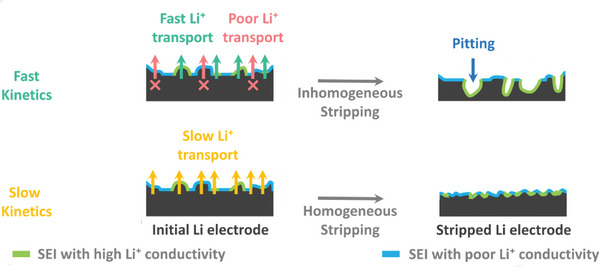
Schematic illustration of stripping processes from Li metal electrode.

**Figure** **4**a,b depict the Nyquist plots derived from the electrochemical impedance spectra (EIS) of Li|Li symmetric cells immersed in LiTFSI and LiNO₃‐based electrolytes, respectively. These impedance results were analyzed using an equivalent circuit (as described in the Supporting information experimental section) to extract relevant impedance parameters, as shown in Figure 4c–f. Comparing Figure 4c,e, it is evident that the interphasial impedances (R_SEI_) in LiTFSI‐based electrolytes are significantly higher than those in LiNO₃. This discrepancy may be attributed to the increased decomposition of LiTFSI‐based electrolytes and the accumulation of more insulating compounds on the electrode surface, leading to higher R_SEI_ values. Interestingly, Figure 4d,f shows a clear trend of decreasing charge transfer resistance (R_CT_) as the glyme chain length decreases for both LiTFSI and LiNO₃. This observation suggests that as the glyme chain length decreases, the charge transfer kinetics improves, and the propensity of preferential Li stripping from regions beneath the “hot spots” leading to pit formation also increases. Consequently, an increase in surface roughness is observed in Figure 2b as the glyme chain length decreases. A mechanistic explanation of the different stripping processes is schematically shown in **Figure** **5**.

#### 2.3.2 Plating on Cu

**Figure 6:**
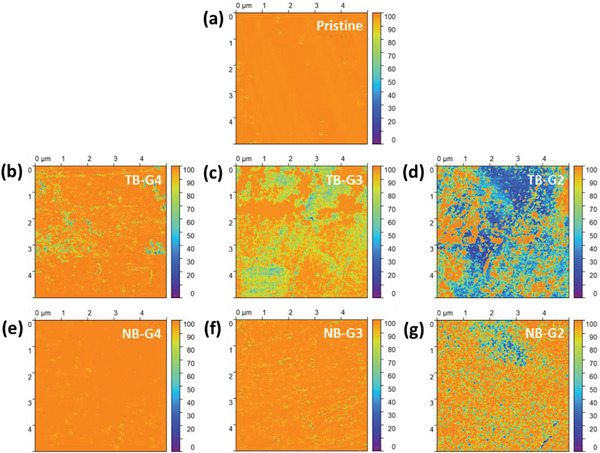
Electronic conductivity distribution measured by peak force tunneling AFM (PFTUNA) on Cu at the pristine state (a), and after a galvanostatic sweep from open circuit voltage (OCV) to 0 V versus Li/Li⁺ in (b) TB‐G4, (c) TB‐G3, (d) TB‐G2, (e) NB‐G4, (f) NB‐G3, and (g) NB‐G2. The color scale for the conductivity represents the relative electronic conductivity values on the surface.

**Figure 7:**
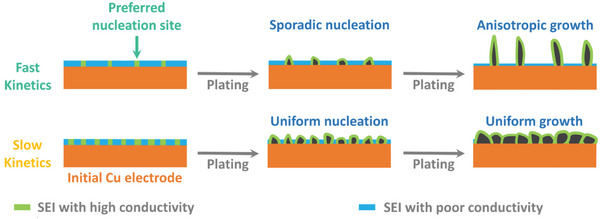
Schematic illustration of Li plating processes on Cu electrode.

**Figure 8:**
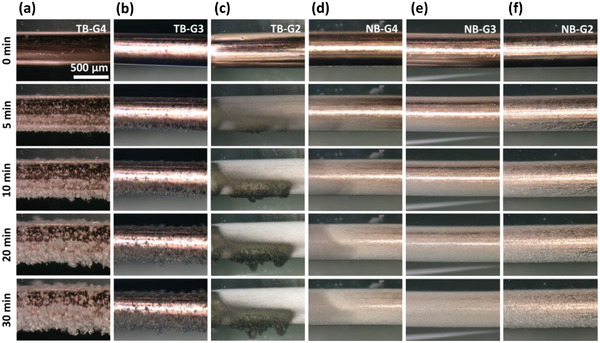
In situ optical micrographs of Li deposition on Cu wire electrodes at selected time intervals in (a) TB‐G4, (b) TB‐G3, (c) TB‐G2, (d) NB‐G4, (e) NB‐G3, and (f) NB‐G2.

The deposition of Li on the Cu electrode reveals a dependency on both the Li salt and the glyme solvent. Specifically, longer glymes and LiNO₃ as the electrolyte salt inhibit dendritic/whisker‐like Li deposition. Despite the initially flat surface of the Cu electrode, significant variations in deposition morphologies underscore the influence of electrolyte properties. The trends observed in Figure 4d,f indicate slower charge transfer kinetics with increasing glyme chain length and the use of LiNO₃ as the electrolyte salt. This deceleration in kinetics, leading to more stable Li deposition, agrees well with previous findings.^[^ ^41^ ^]^ Low charge transfer resistance (or high exchange current density) during Li deposition often results in an uneven distribution of local current density, leading to preferred deposition on areas with higher local current density.^[^ ^53^ ^]^ Moreover, according to a previous phase‐field model for metal deposition, faster kinetics causes higher current density at the deposit tip.^[^ ^54^ ^]^ These sporadic nucleations and preferred deposition at the tip under faster kinetics subsequently trigger anisotropic growth. In contrast, higher charge transfer resistance helps mitigate the uneven distribution of current density, promoting more homogeneous nucleation and growth of Li. However, the spatial variation in deposited Li morphologies on Cu in TB‐G2 and TB‐G3 cannot be solely attributed to deposition kinetics. One possibility is the physicochemical inhomogeneity on the Cu surface. Nevertheless, obtaining XPS at multiple locations also fails to provide a comprehensive understanding of this inhomogeneity with sufficient spatial resolution. Instead, we measured the electronic conductivity distribution on the Cu electrode using peak force tunneling atomic force microscope (PFTUNA) after a galvanostatic sweep of Li|Cu cells in different electrolytes from the open circuit voltage (OCV) to 0 V versus Li/Li⁺. During the galvanostatic sweep to 0 V versus Li/Li⁺, the Cu compounds on the surface are reduced, and an electrolyte‐dependent surface film is formed.^[^ ^55^ ^]^ Due to the differences in reduction propensities, unique characteristics are expected in surface films formed by different electrolytes. **Figure** **6**a depicts a relatively homogeneous current distribution on pristine Cu. However, after the galvanostatic sweep, an increase in inhomogeneity is observed in any electrolyte, except for NB‐G4. In LiTFSI‐based electrolytes (Figure 6b–d), the inhomogeneity is more pronounced, which further intensifies as the glyme size decreases. XPS results in Figure 3 indicate that the electrolyte decomposition products in TB‐G2, TB‐G3, and TB‐G4 are primarily Li₂O and Li₂CO₃, which are insulators with wide bandgaps.^[^ ^56^, ^57^ ^]^ Consequently, these insulating compounds are also expected to accumulate on the Cu surface, causing spatial variations in electronic conductivity. Similar explanation applies to the observed inhomogeneous current distribution with NB‐G2. In contrast, the relatively uniform current distributions with NB‐G3 and NB‐G4 can be attributed to limited electrolyte decomposition after the voltage sweep up to 0 V versus Li/Li⁺. Therefore, due to the low reductive stabilities, the accumulation of insulating compounds resulting in highly inhomogeneous electronic conductivity distribution in LiTFSI‐based electrolytes, especially in TB‐G2 and TB‐G3, leads to sporadic nucleation and growth, and spatially variable Li morphologies. Conversely, the more uniform surface (Figure 6e–g) and relatively slower kinetics in LiNO₃‐based electrolytes promote spherical Li deposition across the electrode surface. In fact, the slower kinetics appears to mitigate the effects of observed inhomogeneity on the Cu surface in NB‐G2. **Figure** **7** illustrates a schematic explanation of Li deposition exhibiting various morphologies on the Cu electrode surface. The variations in nucleation and growth patterns of Li in different electrolytes were directly observed in situ using an optical microscope. A schematic illustration of the cell employed for these in situ optical measurements is provided in Figure S23 (Supporting Information). Snapshots captured at specific time intervals during Li deposition on a Cu electrode are presented in **Figure** **8**a–f. The images clearly depict the uneven nucleation and growth of mossy and whisker‐like Li structures in LiTFSI‐based electrolytes. Although the precise growth location of an individual whisker cannot be directly observed at the current magnification, a holistic assessment confirms that the porous Li deposit extends outward from its base. Moreover, the differences in glyme length‐dependent stability of the electrolyte with the deposited Li is to some extent evident through the in situ microscopic experiments. The Li deposited in the TB‐G4 electrolyte exhibits a distinct metallic luster. In contrast, the TB‐G2 and TB‐G3 electrolytes result in a dark grey deposit. These observations suggest that TB‐G2 and TB‐G3 exhibit higher reactivity towards the deposited Li. Conversely, in LiNO₃‐based electrolytes, the nucleation and growth of Li appear more homogeneous and compact, irrespective of the glyme solvent used.

#### 2.3.3 Plating on Previously Stripped Li

**Figure 9:**
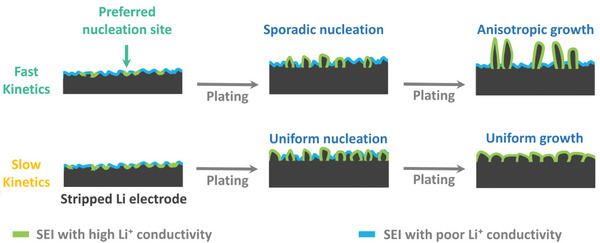
Schematic illustration of Li plating processes on previously stripped Li electrode.

The same interpretations that explained Li deposition on Cu electrodes apply to understanding the Li deposition morphologies on a previously stripped Li surface. Increased inhomogeneity during stripping and higher charge transfer kinetics with shorter chain glymes and LiTFSI highlight a trend toward inhomogeneous nucleation and unstable whisker‐like Li growth. In contrast, NB‐G3 and NB‐G4 exhibit spherical deposits due to a more uniform interphase and slower deposition kinetics. However, using G2 as the solvent in NB‐G2 leads to both whisker‐like and spherical Li morphologies due to enhanced inhomogeneity in the interphase and higher kinetics. **Figure** **9** depicts a schematic of the Li deposition process on the previously stripped Li electrode.

## 3 Discussion

During Li deposition, the electron transfer process occurs between the Li surface and solvated [Li(G*_n_*)*_x_*]⁺ ions within the electrochemical double layer, leading to potential interfacial electrochemical Reaction (1):

(1) $$\left\{Li_{surface}|{\left[Li{\left(G_{n}\right)}_{x}\right]}^{+}\right\}+e^{-}↔Li_{surface}+Li_{bulk}+xG_{n}$$

**Figure 10:**
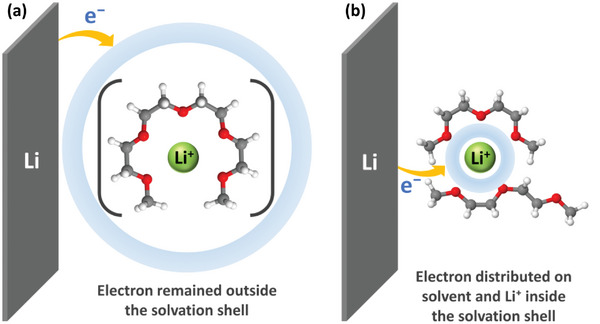
Schematic illustration of the differences in electron distribution in the solvated Li⁺‐ions during reduction depending on the solvation structure in (a) G4 and (b) G2.

Here, G represents glyme ether and *n* represents the number of ethylene moieties in the glyme. In reality, the solvents and anions present in the double layer also undergo reduction, resulting in the formation of the SEI. The reductive stability of the electrolyte dictates the SEI properties, and the spontaneous decomposition of highly unstable electrolytes leads to a thick SEI composed of insulating Li compounds with spatial heterogeneity. When electrons are transferred from the electrode surface to solvated species, the transferred electron can reside on the Li⁺ center, the attached solvents/anions, or the exterior of the solvation sphere.^[^ ^58^ ^]^ Previous studies have shown that this electron distribution depends significantly on the solvation structure of the Li⁺ ions.^[^ ^58^, ^59^, ^60^ ^]^ In this context, glyme ethers offer a distinctive solvation environment for Li⁺ ions due to the chelation capability of glymes through multiple coordination sites. From a coordination chemistry standpoint, the chelate effect produced by multidentate ligands provides increased stability to the complex ions; therefore, longer glymes create more stable complexes with Li⁺ ions.^[^ ^35^, ^61^ ^]^ While the absolute values of the total formation energies for the [Li(G*_n_*)*_x_*]⁺ complexes increase as the glyme chain length increases due to the growing number of Li∙∙∙O contacts, previous research has demonstrated that, for a 1:1 complex, the formation energy per Li∙∙∙O contact decreases.^[^ ^62^ ^]^ A stronger interaction between the Li⁺‐ion and the O atom of the glyme results in the coordinating solvent being more polarized, more electrophilic, and more susceptible to reduction. Density functional theory (DFT) calculations suggest that in cases of smaller glymes, the transferred electron is distributed within the ionic radius of the metal center and on the organic part of the solvation sphere.^[^ ^58^ ^]^ This indicates competition between metal ion reduction and solvent decomposition, with electron transfer occurring via an outer sphere pathway without breaking the primary solvation sheath. Conversely, longer glymes like G4 result in electron accumulation in the second solvation sphere, indicating that the electron transferred from the metal surface stays outside the solvation shell without being instantaneously accepted within the solvation shell of the metal ion. A qualitative description of the distribution of the transferred electron in different solvated Li⁺ ions is shown in **Figure** **10**. This comparison underscores the significant impact of glyme chain length on electrode reaction mechanisms. With shorter glyme lengths, solvent reduction propensity increases, and electrode reaction kinetics accelerate, primarily limited by the electron transfer step. Conversely, for the longer glymes, the increasing chelation around the Li⁺ ions effectively shields the outer s‐orbital from interaction with the electron.^[^ ^59^ ^]^ This strong solvation by chelation requires partial desolvation to facilitate metal ion reduction, retarding the metal reduction kinetics and minimizing electron transfer to associated solvents.^[^ ^58^ ^]^

Applying this understanding to our system suggests that as glyme chain length decreases from G4 to G2, electrolyte reductive stability declines, facilitating enhanced electrolyte reduction and subsequent formation of thick, heterogeneous SEI layers containing insulating Li compounds on the electrode surface. Apparently, this enhanced stability of the electrolyte due to higher solvation in longer glymes seems contradictory to the large number of reports favoring solvents with weaker solvation strength to exclude the solvents from the primary solvation shells and induce contact ion pairs or aggregated structures with the anion.^[^ ^27^, ^63^ ^]^ In such electrolytes, the lowest unoccupied molecular orbital (LUMO) typically shifts from the solvent to the anion, avoiding solvent decomposition at the expense of anion decomposition. In contrast, we compare the relative stabilities of different glyme solvents under the completely solvated conditions of Li⁺ ions at a relatively lower salt concentration of 1 m, where the solvents are expected to be predominantly present in the primary solvation shell. The results demonstrate that under these solvated conditions, longer glymes with a unique solvation structure due to chelation provide better stability to the electrolyte. Current measurements using PFTUNA on the reduced Cu electrode surface revealed, in Figure 6, higher inhomogeneity with smaller glyme electrolytes due to the accumulation of insulating electrolyte decomposition products. These surface inhomogeneities lead to non‐uniform stripping/plating of Li from/on the Li/Cu electrode at favored sites where there is adequate Li⁺ ion conductivity through the SEI, leading to sporadic pitting/nucleation. Furthermore, higher charge transfer kinetics (Figure 4d,f) in smaller glyme electrolytes exacerbate the inhomogeneous stripping/plating. However, similar to previous reports, when employing LiNO₃ as the electrolyte salt, its highly associated nature results in the retention of NO₃⁻ ions in the primary solvation shell as contact ion pairs, and these ions predominantly occupy the inner Helmholtz plane (IHP) on the Li electrode.^[^ ^64^ ^]^ Consequently, LiNO₃‐based electrolytes lower the feasibility of solvent decomposition.^[^ ^65^ ^]^ Lower R_SEI_ values in LiNO₃‐based electrolytes also provide evidence of a relatively smaller extent of electrolyte decomposition on the electrode surface. Furthermore, LiNO₃ reduces charge transfer kinetics compared to LiTFSI, as observed in Figure 4d compared to Figure 4f. The combined effects of these phenomena promote stable Li stripping/plating in LiNO₃‐based electrolytes. Therefore, the differences in solvation structure based on glyme chain length and electrolyte salt are considered the underlying factors contributing to the differences in electrolyte stability, interphasial properties, and deposition/dissolution kinetics. These variations impact the morphologies of Li deposition and the cycle life of high‐capacity cells.

## 4 Conclusion

In summary, we have explored the impact of glyme chain length on the stability of Li metal electrode and the cycle life of high areal‐capacity LMB cells with limited electrolyte volume. To gain insights into the morphological changes during Li stripping and plating, we investigated the physicochemical properties of the SEI and the interphasial kinetics of the stripping/plating reaction across different electrolytes. Our electrolyte compositions included three glymes with varying chain lengths – diglyme, triglyme, and tetraglyme – combined with 1 m LiTFSI or LiNO₃ salts and 0.05 m LiBr as an additive. We observed that reducing the glyme chain length increased both interphasial kinetics and SEI inhomogeneity, particularly pronounced with LiTFSI as the electrolyte salt. Consequently, smaller glymes and LiTFSI promoted unstable stripping and plating, resulting in pitting and dendritic Li deposition. Conversely, longer glymes (G3 and G4) with LiNO₃ salt exhibited more uniform stripping and compact spherical Li deposition. These variations in interphasial properties, Li deposition morphologies, and cycle life are attributed to differences in solvation structure dependent on glyme chain length and electrolyte salt. According to previous density functional theory (DFT) calculations, smaller glymes with weak cation solvation undergo competition between metal ion reduction and solvent decomposition, leading to electron transfer with facile kinetics via an outer sphere pathway without breaking the primary solvation sheath. Conversely, stronger solvation with longer glymes like G4 does not allow the electron to enter the primary solvation shell instantaneously and requires a partial desolvation to facilitate metal ion reduction, slowing down the kinetics and also minimizing electron transfer to associated solvents. Additionally, LiNO₃‐based electrolytes retain NO₃⁻ ions in the primary solvation shell as contact ion pairs, primarily occupying the inner Helmholtz plane (IHP) on the Li electrode, thereby reducing solvent decomposition feasibility. The slower kinetics and favorable SEI properties promote stable Li stripping/plating in longer glymes and LiNO₃‐based electrolytes. Nonetheless, while slower kinetics favor stable Li deposition, there is an expected trade‐off between morphological stability and voltage polarization due to the large charge transfer resistance (slow kinetics), especially at high rates of stripping/plating. This trade‐off warrants further attention and more detailed investigation. The results presented in this study underscore the significant impact of glyme chain length on the electrochemical processes at Li metal electrodes.

## Conflict of Interest

The authors declare no conflict of interest.

## Supporting information

Supporting Information

## Data Availability

The data that support the findings of this study are available from the corresponding author upon reasonable request.
