# Supplementary material for: Impact of Glyme Ether Chain Length on the Interphasial Stability of Lithium‐Electrode in High‐Capacity Lithium‐Metal Battery
Source: Adv Sci (Weinh). 2024 Jun 21;11(32):2404245. doi: 10.1002/advs.202404245 (PMC11348056; doi:10.1002/advs.202404245)
Supplement: Supplementary file 1 — Supporting Information [file ADVS-11-2404245-s001.pdf]

ADVANCED SCIENCE
Open Access

# Supporting Information

for *Adv. Sci.*, DOI 10.1002/advs.202404245

Impact of Glyme Ether Chain Length on the Interphasial Stability of Lithium-Electrode in High-Capacity Lithium-Metal Battery

*Arghya Dutta*, Kyosuke Matsushita and Yoshimi Kubo**

# Supporting Information

## Impact of Glyme Ether Chain Length on the Interphasial Stability of Lithium-Electrode in High-Capacity Lithium-Metal Battery

Arghya Dutta*, Kyosuke Matsushita and Yoshimi Kubo*

### 1. Experimental section

### 1.1. Electrolyte preparation

High-purity tetraethylene glycol dimethyl ether (tetraglyme or G4), triethylene glycol dimethyl ether (triglyme or G3), and diethylene glycol dimethyl ether (diglyme or G2) with a water content of less than 30 ppm were obtained from Japan Advanced Chemicals and used without additional purification. The electrolyte salts, lithium bis(trifluoromethanesulfonyl)imide (LiTFSI; KISHIDA CHEMICAL Co., Ltd.), anhydrous lithium nitrate ($LiNO_3$; KISHIDA CHEMICAL Co., Ltd.), and anhydrous lithium bromide (LiBr; Sigma-Aldrich) were dried at 120 °C under vacuum for 24 hours inside a super dry-room (dew point < −60 °C) before the preparation of the electrolytes. All electrolytes were prepared within an argon (Ar) filled glovebox (dew point < −90 °C).

### 1.2. Electrochemical cells

Stacked Li|Copper (Cu) and Li|Li cells (Figure S1) were employed to investigate Li stripping/plating. The stack consisted of either a Li metal (16 mm diameter, 0.2 mm thickness; Honjo Metal) and a Cu foil (19 mm diameter, 0.01 mm thickness; Nilaco) or two Li metal foils separated by a Celgard® 2325 separator soaked in the electrolyte (50 µL). Assembly of all cells was conducted within an Ar-filled glovebox (dew point < −90 °C). For in situ optical microscopic observation of Li plating on a Cu wire (0.5 mm diameter, Nilaco), a transparent glass tube cell (Figure S16) was utilized. The separation between the Li and Cu electrodes was maintained at 5 mm. The electrodes under observation were plated at a current density of 0.2 mA $cm^{-2}$.

The Li–$O_2$ cells were assembled inside an argon-filled glovebox (dew point < -90 °C) using flow-type cells (Figure S1) with a continuous oxygen flow (~20 mL $min^{-1}$). The Li–$O_2$ cells consisted of a Li metal negative electrode (1.6 cm in diameter, 0.2 mm thickness, Honjo Metal), a Celgard® 2325 separator (1.9 cm in diameter), a glass fiber (GF/C, 1.6 cm in diameter, Whatman®), and a positive electrode (1.6 cm in diameter) made of ketjenblack (EC600JD, Lion, 1 mg $cm^{-2}$) coated on carbon paper (TGP-H-30, Toray). The non-aqueous electrolytes (100 μL) were composed of 1 M $LiNO_3$ + 0.05 M LiBr dissolved in different glyme ethers (G2, G3, and G4). The cells were cycled at a current density of 100 mA $g^{-1}$ with a fixed capacity of 500 mAh $g^{-1}$. The positive electrode was replaced and the electrolyte was replenished whenever the capacity dropped below 500 mAh $g^{-1}$, while the same negative electrode and separators were retained throughout the cycling.

**1.3. Measurements and characterizations**

All the galvanostatic Li stripping/plating processes were carried out using an electrochemical tester by Hokuto Denko, HJ1001SD8. The electrochemical impedance spectra (EIS) of Li|Li symmetric cells were measured after a stripping/plating cycle (4 mAh $cm^{-2}$ at 0.2 mA $cm^{-2}$) by Biologic VSP potentiostat/galvanostat in the frequency range $10^6$ to 0.005 Hz under potentiostatic mode, with an amplitude of 10 mV. The EIS data were fitted with the equivalent circuit: $(R_{Ohmic})-(R_{SEI}Q)-(R_{CT}Q)$. The conductivities of the electrolytes were measured by Mettler Toledo, SevenExcellence. Morphology of the electrodes were observed on a JSM-7800F field-emission scanning electron microscope (FE-SEM, JEOL) and chemical compositions of the interphase were analyzed on an energy dispersive X-ray spectrometer (X-MaxN 50, Oxford) fitted with the SEM. In situ Li stripping/plating was observed by a Keyence VHX digital microscope. X-ray photoelectron spectra (XPS) of the electrodes were recorded on a VersaProbe II Scanning XPS Microprobe (ULVAC-PHY) coupled with argon gas cluster ion beam (Ar-GCIB). All the experiments were carried out either inside an Ar filled glovebox

(dew point < −90 °C) or in a super dryroom (dew point < −60 °C). Surface electronic conductivity distribution was measured using an Atomic Force Microscope (AFM; Bruker AXS, Multimode 8) operating in Peak Force Tunneling AFM (PFTUNA) mode. The AFM was placed within an Ar-filled glovebox ($H_2O$ and $O_2$ < 1 ppm). A PFTUNA conductive probe with a spring constant of 0.4 N $m^{-1}$ and a tip radius of 20 nm was utilized for this experiment. The scanned sample area measured 5 µm × 5 µm and the scanning was performed at a consistent rate (0.2 Hz) with a bias set at 0.5 V. For SEM, XPS and PFTUNA measurements hermetically sealed transfer vessels were used.

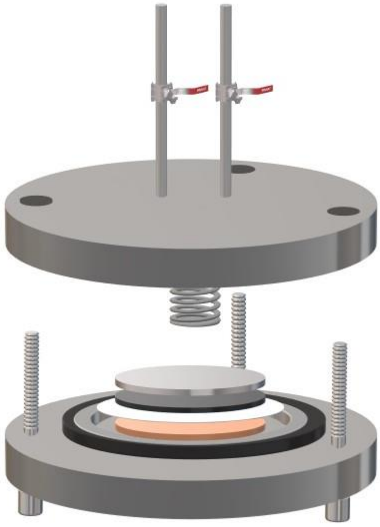

**Figure S1.** Schematic representation of the cell used in this work. From top to bottom, the cell components are stainless steel current collector, Li metal, Celgard® 2325 separator and Cu metal.

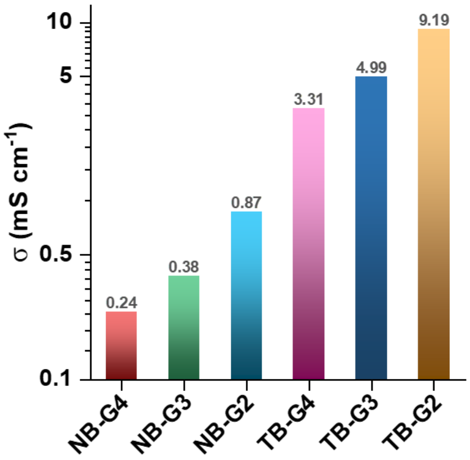


**Figure S2**. Ionic conductivities of different electrolytes measured at 25 °C.

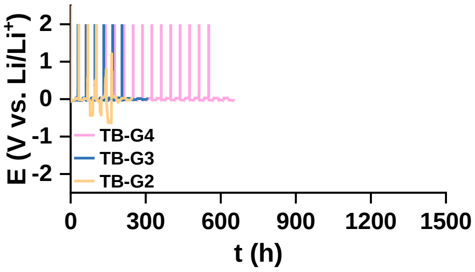


**Figure S3**. Voltage versus time curves of Li stripping/plating in Li|Cu cells at 0.2 mA $cm^{-2}$ current density using LiTFSI-based electrolytes.

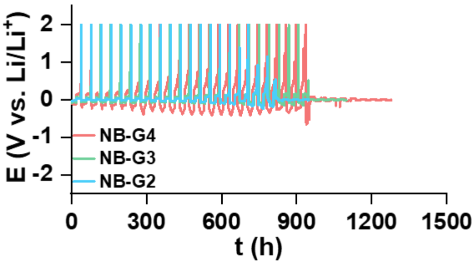


**Figure S4**. Voltage versus time curves of Li stripping/plating in Li|Cu cells at 0.2 mA $cm^{-2}$ current density using $LiNO_3$-based electrolytes.

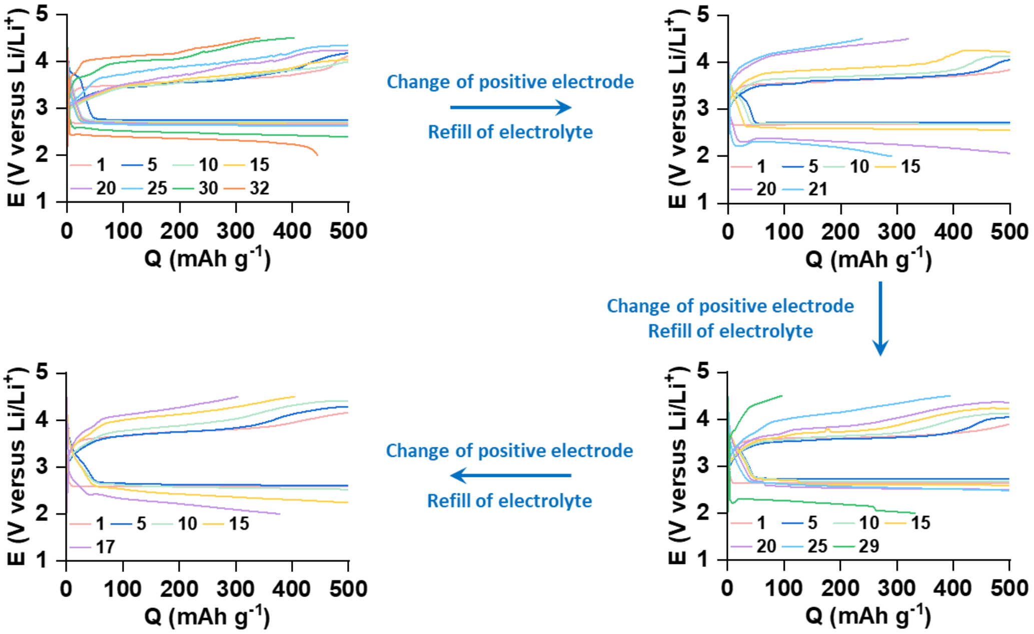


**Figure S5**. Galvanostatic discharge/charge curves of Li-$O_2$ cell with ketjenblack electrode at a current density of 100 mA $g^{-1}$ with a fixed capacity of 500 mAh $g^{-1}$ in NB-G4 electrolyte. The positive electrode of the cell was replaced with a fresh one and the electrolyte was replenished every time the capacity fell below 500 mAh $g^{-1}$.

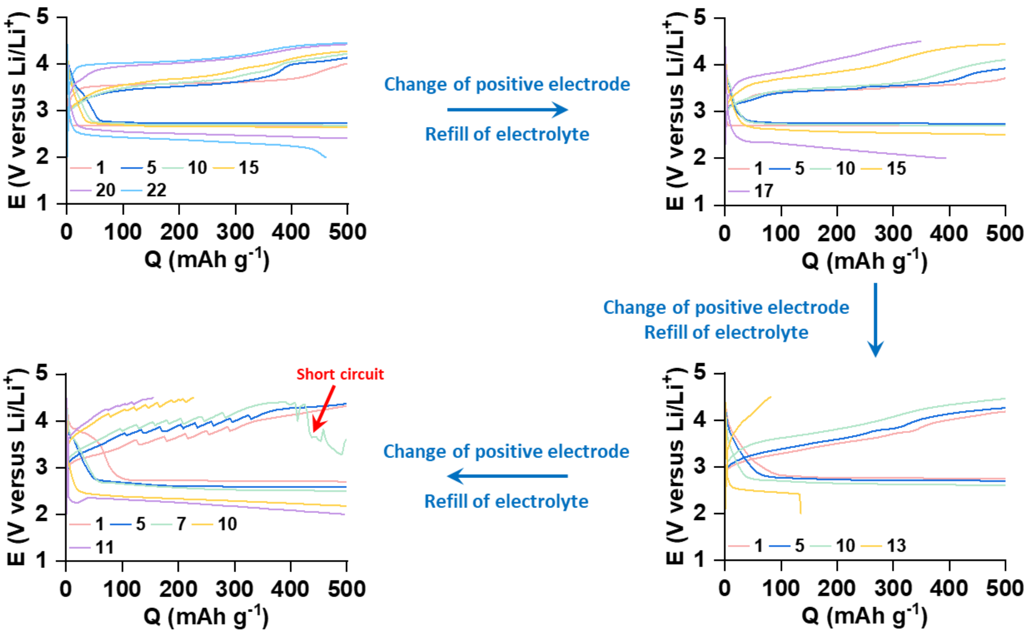


**Figure S6**. Galvanostatic discharge/charge curves of Li-$O_2$ cell with ketjenblack electrode at a current density of 100 mA $g^{-1}$ with a fixed capacity of 500 mAh $g^{-1}$ in NB-G3 electrolyte. The positive electrode of the cell was replaced with a fresh one and the electrolyte was replenished every time the capacity fell below 500 mAh $g^{-1}$.

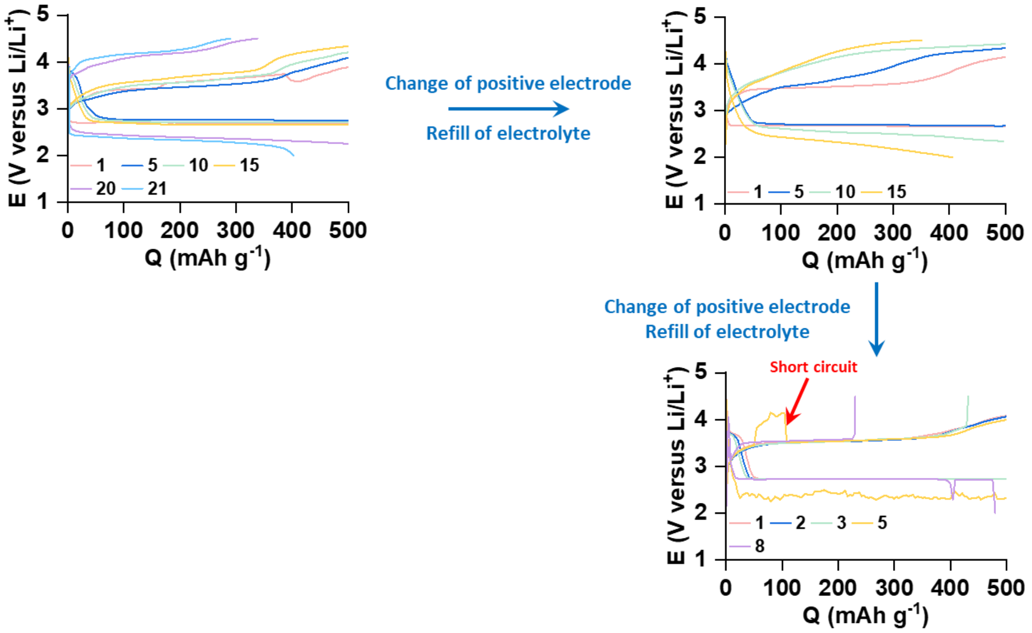


**Figure S7**. Galvanostatic discharge/charge curves of Li-$O_2$ cell with ketjenblack electrode at a current density of 100 mA $g^{-1}$ with a fixed capacity of 500 mAh $g^{-1}$ in NB-G2 electrolyte. The positive electrode of the cell was replaced with a fresh one and the electrolyte was replenished every time the capacity fell below 500 mAh $g^{-1}$.

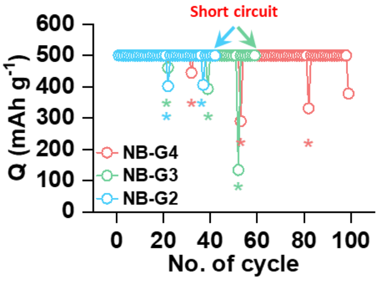


**Figure S8**. Capacity versus cycle life plot for Li-$O_2$ cell with ketjenblack electrode at a current density of 100 mA $g^{-1}$ with a fixed capacity of 500 mAh $g^{-1}$ in NB-G2, NB-G3, and NB-G4 electrolytes. The positive electrodes of the cells were replaced with a fresh one and the electrolyte was replenished every time the capacity fell below 500 mAh $g^{-1}$. The asterisks mark the cycle where the ketjenblack electrode and the electrolyte were replaced.

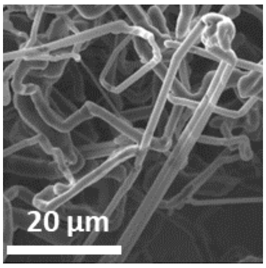


**Figure S9**. Thin whisker-like morphology of deposited Li on Cu surface in TB-G2 electrolyte.

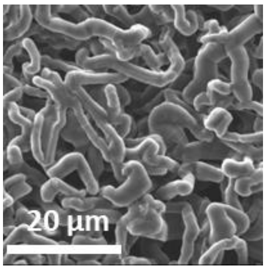


**Figure S10**. Thicker whisker-like morphology of deposited Li on Cu surface in TB-G2 electrolyte.

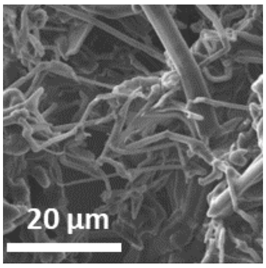


**Figure S11**. Whisker-like morphology of deposited Li on Cu surface in TB-G3 electrolyte.

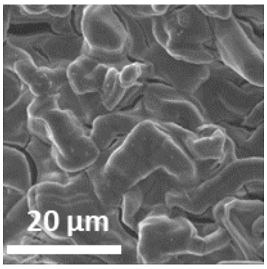


**Figure S12**. Moss-like morphology of deposited Li on Cu surface in TB-G3 electrolyte.

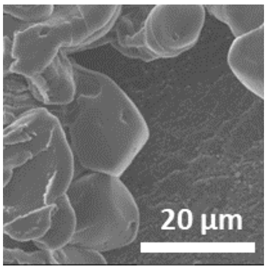


**Figure S13**. Spherical morphology of deposited Li on Li surface in NB-G2 electrolyte.

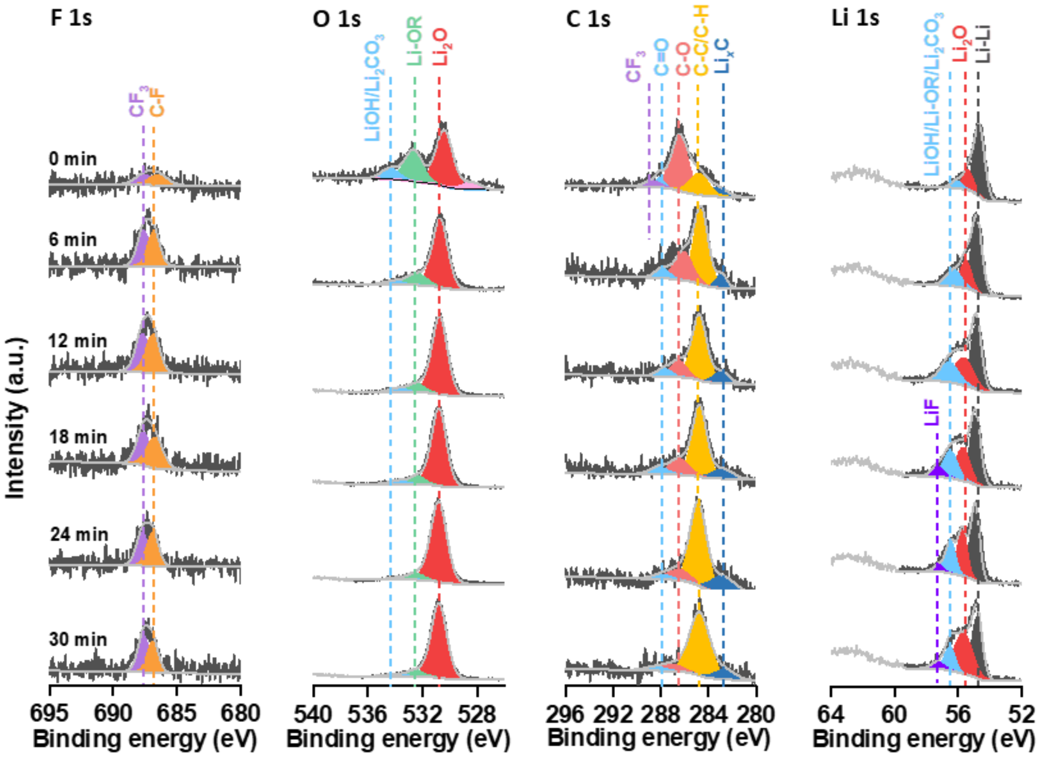


**Figure S14**. X-ray photoelectron spectra for different elements at different depths of Li electrode stripped in TB-G4.

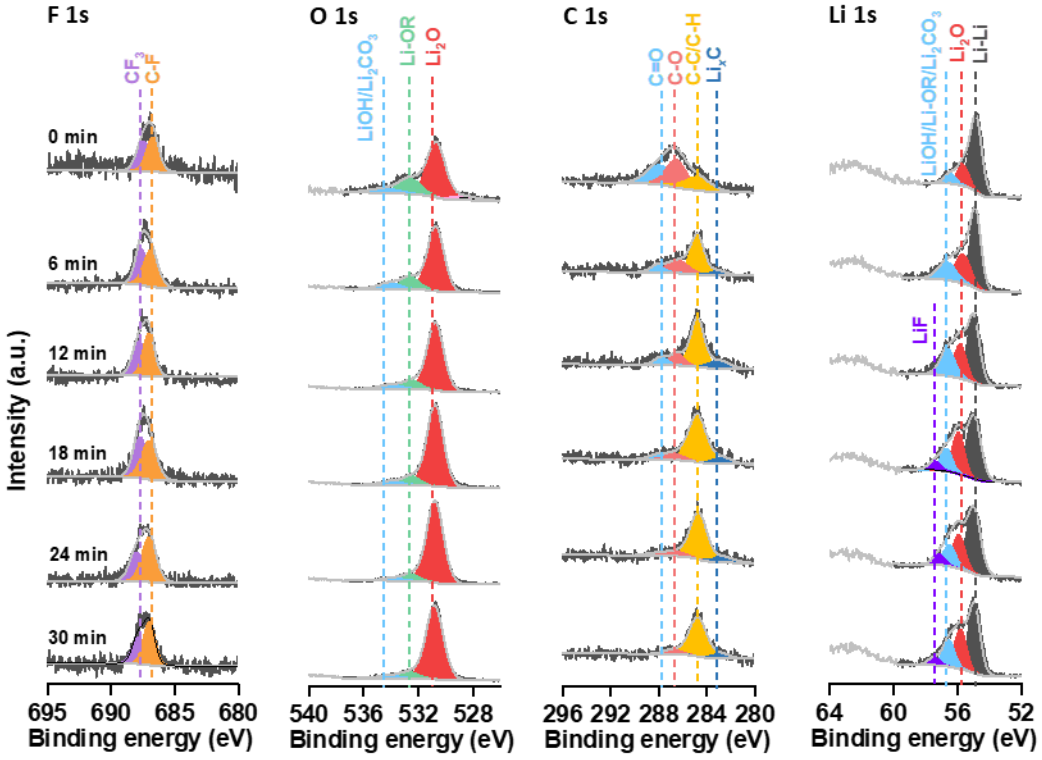


**Figure S15**. X-ray photoelectron spectra for different elements at different depths of Li electrode stripped in TB-G3.

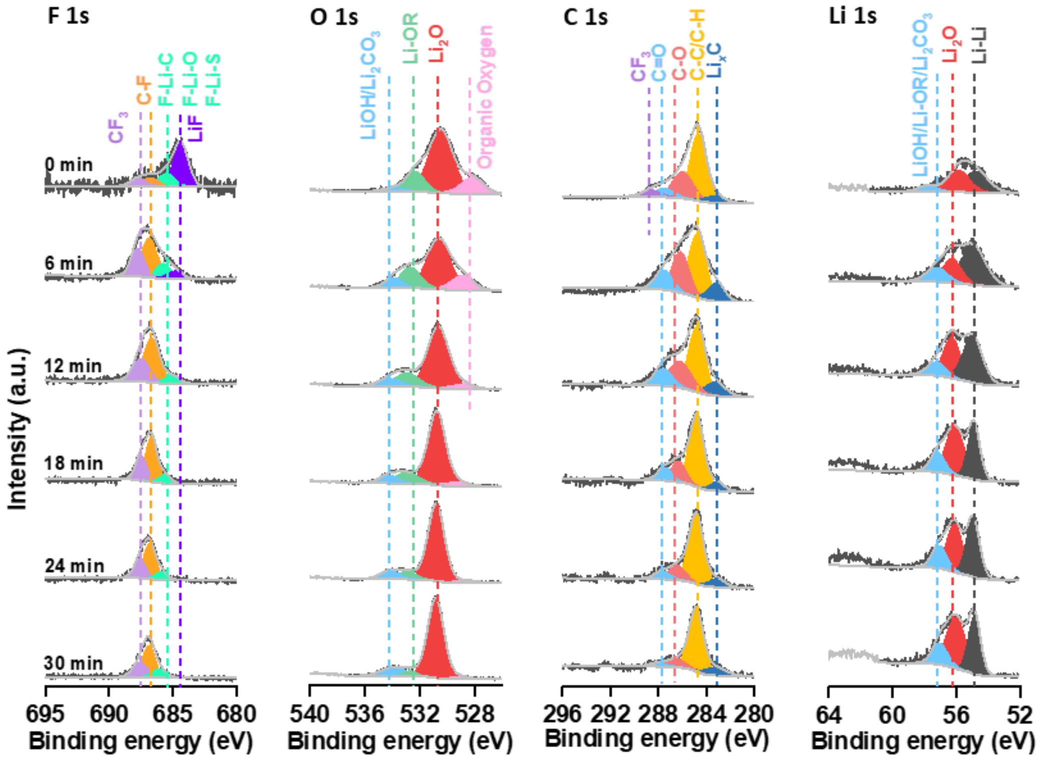


**Figure S16**. X-ray photoelectron spectra for different elements at different depths of Li electrode stripped in TB-G2.

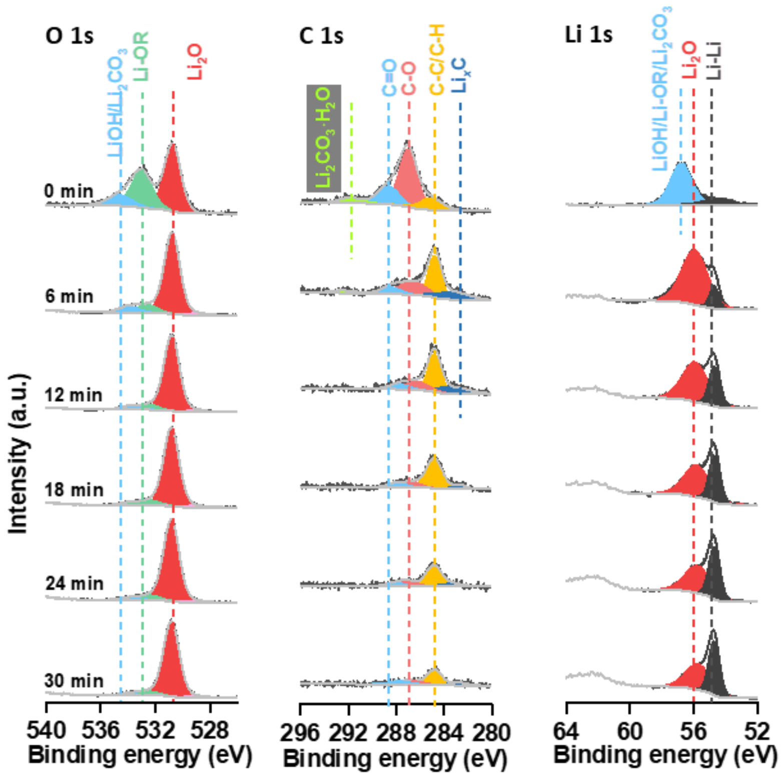


**Figure S17**. X-ray photoelectron spectra for different elements at different depths of Li electrode stripped in NB-G4.

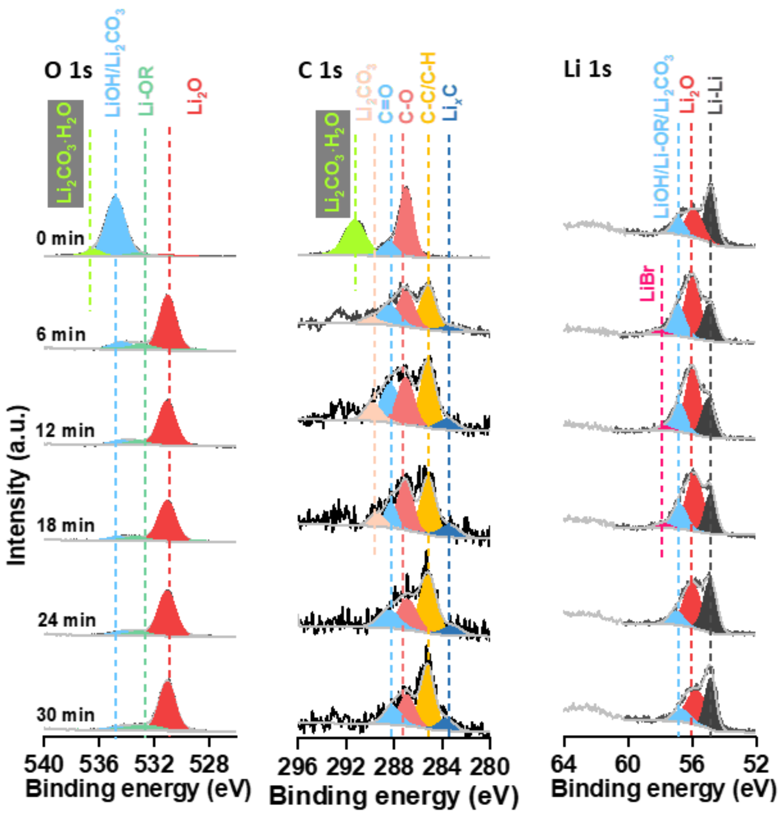


**Figure S18**. X-ray photoelectron spectra for different elements at different depths of Li electrode stripped in NB-G3.

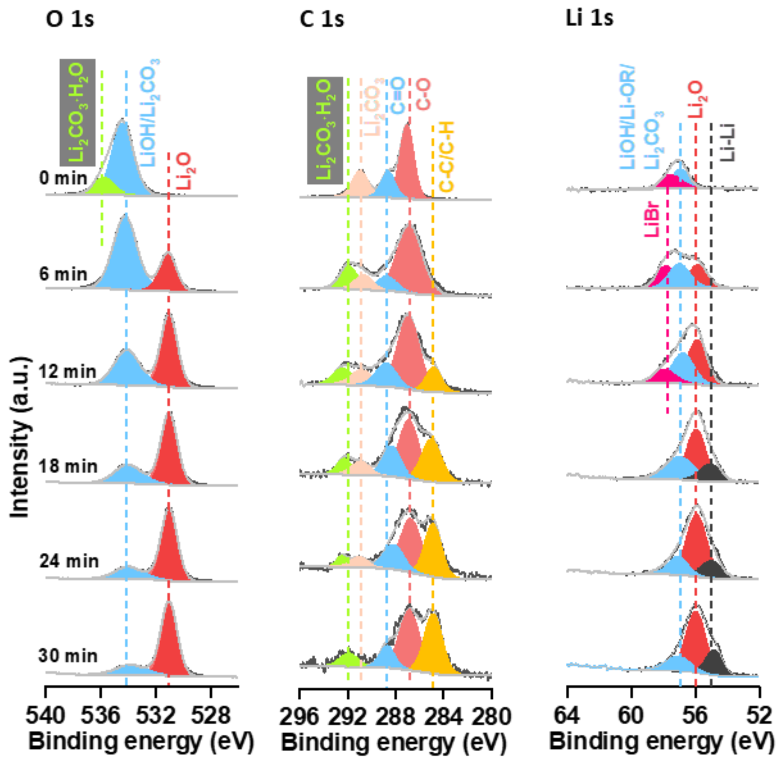


**Figure S19**. X-ray photoelectron spectra for different elements at different depths of Li electrode stripped in NB-G2.

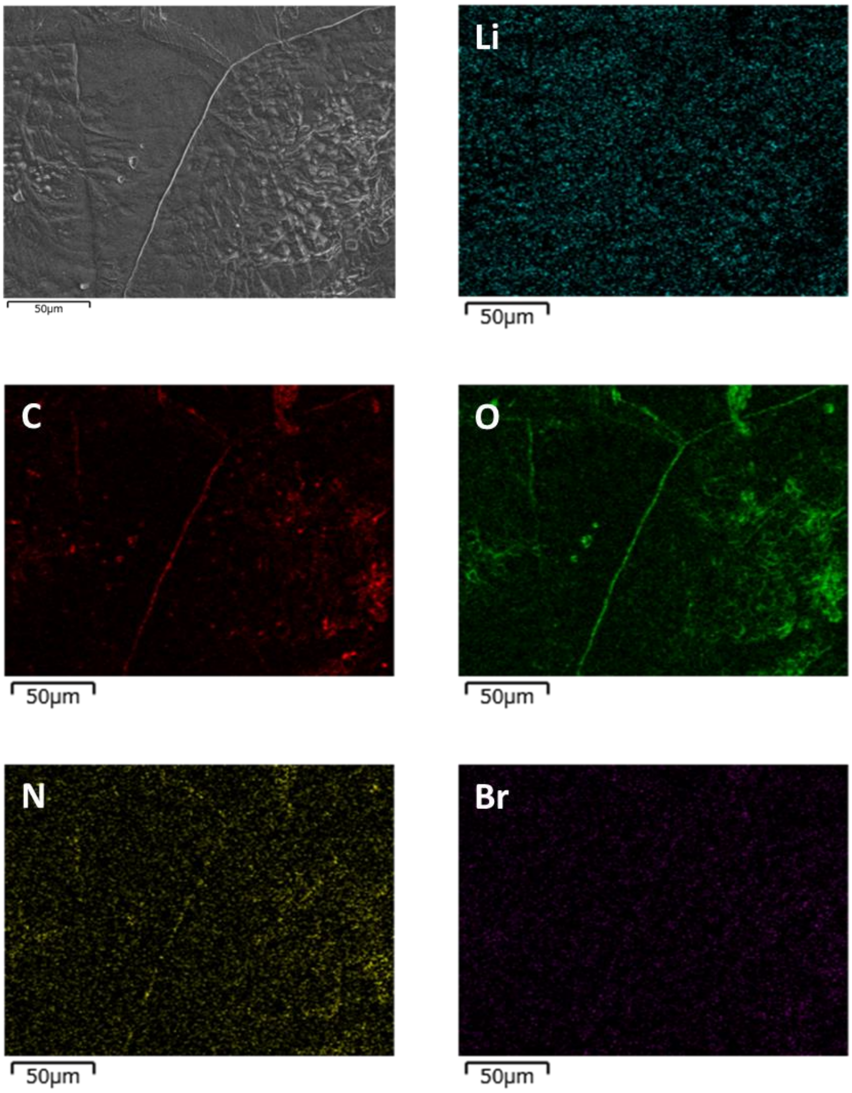


**Figure S20**. Energy dispersive X-ray (EDX) mapping of Li electrode after stripping for 4 mAh $cm^{-2}$ in NB-G4.

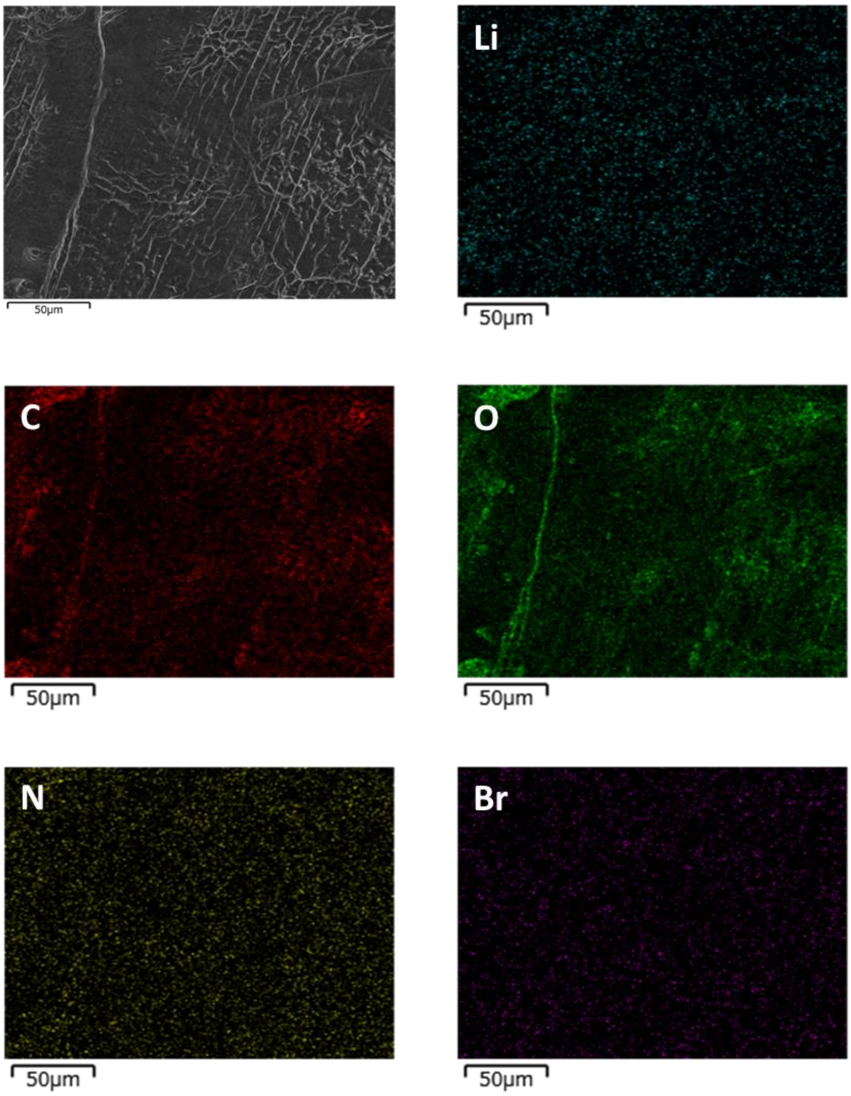


**Figure S21**. Energy dispersive X-ray (EDX) mapping of Li electrode after stripping for 4 mAh $cm^{-2}$ in NB-G3.

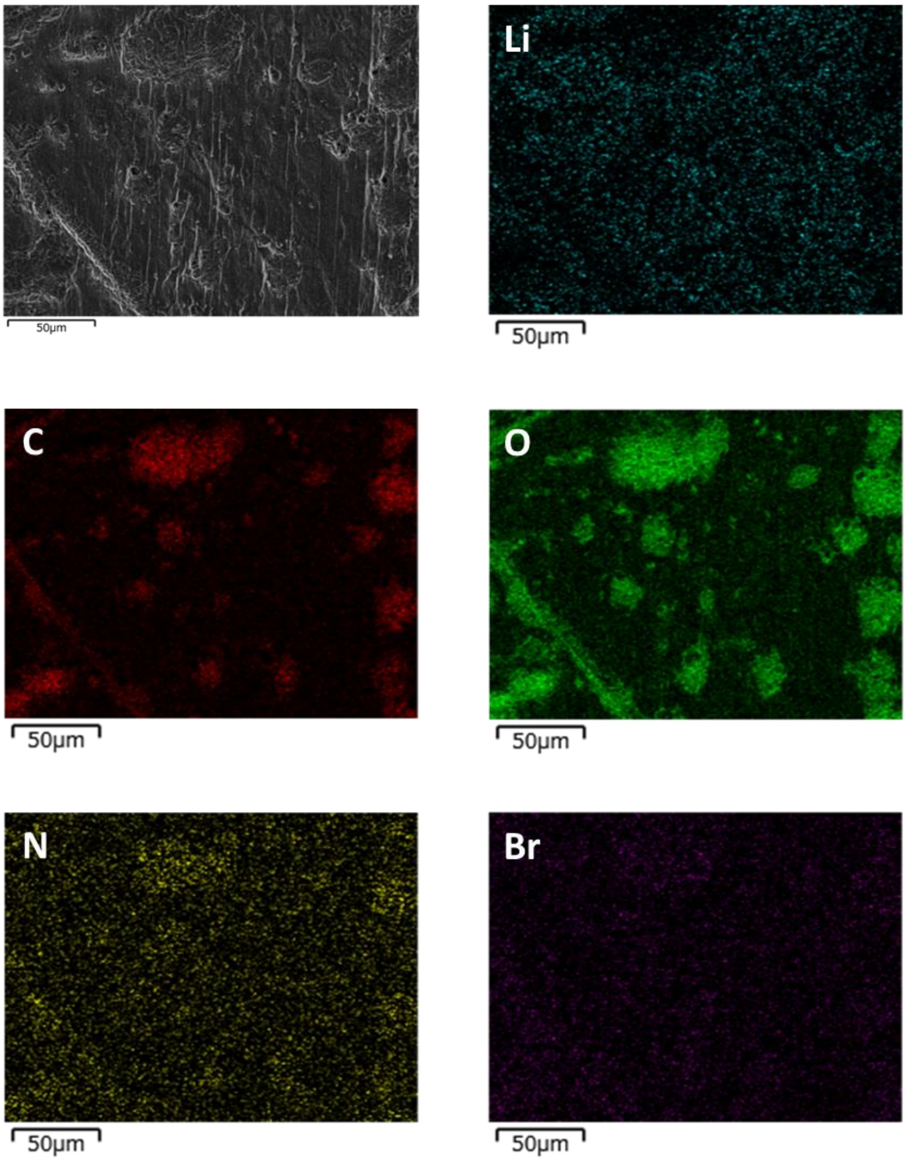


**Figure S22**. Energy dispersive X-ray (EDX) mapping of Li electrode after stripping for 4 mAh $cm^{-2}$ in NB-G2.

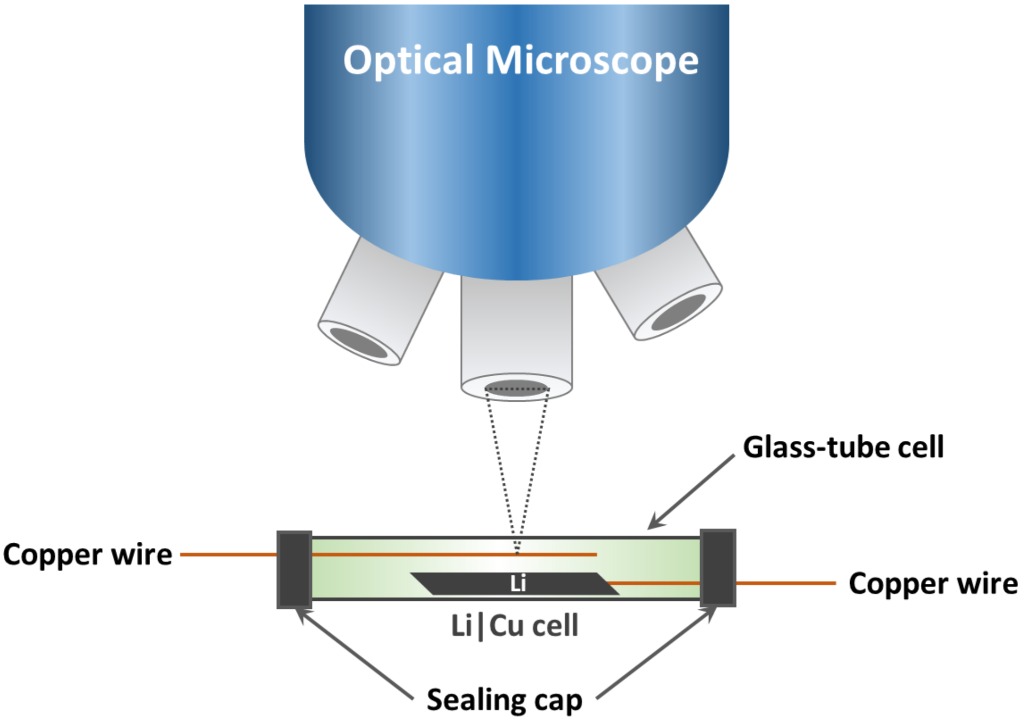


**Figure S23**. Schematic representation of the cell setup used for in situ optical microscopic measurements.

**Table S1**. Quantitative values of different elements estimated by EDX on Li electrodes after stripping in different electrolytes

| Sample | Li (%) | C (%) | O (%) | N (%) | Br (%) |
|---|---|---|---|---|---|
| **NB-G4** | 89.9 | 5.4 | 4.4 | 0.2 | 0.1 |
| **NB-G3** | 84.6 | 8.6 | 6.4 | 0.2 | 0.2 |
| **NB-G2** | 81.2 | 9.6 | 8.9 | 0.2 | 0.1 |
